# Assessment of primary labeling of medicines manufactured by Nepalese pharmaceutical industries

**DOI:** 10.1186/s40545-018-0139-9

**Published:** 2018-06-07

**Authors:** Ramesh Sharma Poudel, Shakti Shrestha, Santosh Thapa, Bhupendra Kumar Poudel, Muniraj Chhetri

**Affiliations:** 1 Hospital Pharmacy, Chitwan Medical College Teaching Hospital, Bharatpur, Chitwan Nepal; 2 Department of Pharmacy, Shree Medical and Technical College, Bharatpur, Chitwan Nepal; 3 Hospital Pharmacy, Ashwins Medical College and Hospitals Pvt. Ltd, Lalitpur, Nepal; 4 Department of Drug Administration, Ministry of Health, Kathmandu, Nepal; 5 School of Public Health, Chitwan Medical College Teaching Hospital, Bharatpur, Chitwan Nepal

**Keywords:** Counterfeit drug, Low-income countries, Labeling, Regulatory standard, Pharmaceutical industry, Nepal, Patient safety

## Abstract

**Background:**

Appropriate labeling of marketed medicines is necessary to fulfill the regulatory provisions and ensure patient medication safety. This study aimed to assess the primary labeling of medicines manufactured and marketed by Nepalese pharmaceutical industries.

**Methods:**

We assessed the primary labeling of all medicines available at the pharmacy of Chitwan Medical College Teaching Hospital (CMCTH), Chitwan, Nepal, between November 2017 to December 2017. Medicines were assessed as required by Drug Standard Regulation, 2043 (1986 AD) of Nepal. Appropriate classification of all the medicines and content of over-the-counter (OTC) medicines (where certain information should be in Nepali language) was also assessed. Descriptive statistics was performed.

**Results:**

Seven hundred fifty-nine medicines manufactured by 37 Nepalese pharmaceutical industries were assessed. While all pharmaceutical products had the name of the drug (brand), only76.8% of them stated drug quantity. Almost all products were found to declare category of the drug, with only a few (4.1%) mentioning the sub-category. The system of medicine was stated in 9.9% of the products. Active ingredients and their quantity, manufacturer’s information, serial number for the production of drug and the date of production, storing methods, and information on the quantity used were mentioned in almost all the products. Similarly, all the products had batch number and the date of expiry. But, 11% of the products lacked the name of pharmacopoeia to which the drug belongs and all the products lacked the serial number for establishment of pharmaceutical industry. Similarly, 5.3% of the products did not list their price, and 2.4% of prescription medicines lacked caution labeling. Unfortunately, the majority of the products (84.4%) did not provide the directions of use. Appropriate drug classification was found in 89.6% of products. None of the over-the-counter medicines totally adhered to the requirements for writing certain information in Nepali language.

**Conclusions:**

Majority of the products did not meet the regulatory standards of primary labeling of Nepalese pharmaceutical products. This study highlights the necessities for improvement from all stakeholders**.**

## Background

Labeling of medicines is a legislative requirement for conveying information about medicines to patients. It provides information on brand, type of drug enclosed within the packaging, strength and quantity of product, appropriate users, instructions, indications, contraindications and side-effects [1]. Therefore, appropriate labeling of medicines is crucial for ensuring patient medication safety, while its ambiguity is associated with administration errors due to incorrect interpretations of products [2]. It has been suggested that medication compliance, distinction, and their readability are affected by medicine labeling that includes design features such as signal word, color, format, wording, language etc. [3]. Evidences demonstrate that marketed pharmaceutical products have, at times, failed to meet the standard labeling requirements [4, 5]. Moreover, studies have also claimed that dispensed pharmaceutical medicines lacked the prescribed labeling requirements [6, 7]. As there are inadequacies in identification and reporting of such requirements in Nepal, Department of Drug Administration (DDA), which is the national medicine regulatory authority, should be more vigilant on such issues [8–10]. In Nepal, allopathic drugs are classified as “a”, “b”, or “c”, with respective sub-categories. Category “a” consists narcotic and poisonous drugs; category “b” consists antibiotics, hormones etc. that require a prescription for dispensing from pharmacies; and category “c” usually consists over-the-counter (OTC) drugs. It has been made obligatory by DDA for pharmaceutical companies to properly label their pharmaceutical products according to the provisions mentioned in Drug Standard Regulation, 2043 (1986 AD) of Nepal [11]. At times, inabilities to follow foreign-language labeling of OTC drugs have resulted in adverse outcomes [12]. To overcome this, DDA has mandated to mention at least name of the drug, directions of use, category/caution, price of the drug, date of production, batch number and expiry date of twenty category “c” medicines in Nepali language [13]. Still then, Nepalese pharmaceutical industries are striving harder to meet the regulatory requirements [14]. It has also been reported that the same generic medicine is placed in two different categories by different pharmaceutical industries [14]. However, the data on this issue is rare in literatures. Therefore, we aimed to assess the primary labeling of medicines manufactured and marketed by Nepalese pharmaceutical industries.

## Methods

Primary labeling of medicines manufactured by Nepalese pharmaceutical industries was assessed between November to December 2017. Only the medicines available at CMCTH, a tertiary hospital located in Chitwan district, Nepal, was assessed. CMCTH was purposefully selected because its pharmacy dispenses most products manufactured by Nepalese Pharmaceutical industries. This hospital only practices allopathic medicines; hence this study only included such medicines. The primary labeling of marketed medicines was assessed as required by Drug Standard Regulation, 2043 (1986 AD) of Nepal [11]. The regulation states that the marketed medicines for human use should contain 16-points information in primary labeling that includes Drug name and quantity; Category and sub category of the drug; System of medicine; Name of active ingredient, quantity and the pharmacopoeia; Name of the drug production company, address and the country; Serial number for the establishment of drug industry; Serial number for the production of the drug; Batch number; Date of production; Date of expiry; Price; Storing methods (techniques) and management of the drug; Caution in Group “a” and “b” (may not be sold without the prescription of a registered medical practitioner); Quantity; Method of use; and additional information on External preparation (only for external use). The primary labeling should contain at least the name of the drug, method of use, category/caution, price, date of production, batch number, and date of expiry of 20 category “c” drugs in Nepali language [13]. A checklist was developed to assess these aforementioned requirements of primary labeling of all kinds of dosage forms. A market basket method (total collection of all available branded generic medicines) was used for sample collection. The appropriateness of classification was also assessed according to Drug Standard Regulation, 2043 (1986 AD) [11]. Descriptive statistics was performed using IBM SPSS version 20 (IBM Corporation, Armonk, NY, USA). The collected generic medicines were categoried according to therapeutic categories and expressed as frequency and percentage.

## Results

This study assessed primary labeling of 759 medicines manufactured by 37 Nepalese pharmaceutical industries. The types of dosage forms assessed are depicted in Table 1.

**Table 1:** Type of dosage form assessed (*n* = 759)

*Dosage form	n(%)
Tablet and Capsule	617(81.3)
Oral Solution, Syrup, Suspension and Drop	65(8.6)
Ointment, Cream and Gel	40(5.3)
Injection	21(2.8)
Nasal Drop	5(0.7)
Eye and Ear Drop	4(0.5)
Mouth Gargle	3(0.4)
External solution and lotion	3(0.4)
Powder	1(0.1)

*Percentage do not add up to 100 due to single decimal place

Of 759 medicines, majority belongs to antimicrobial drugs (20.2%), followed by antihypertensive drugs (13.6%) and vitamins and minerals (8.5%). Details about the therapeutic categories of drugs assessed during study were illustrated in Table 2.

**Table 2:** Classification of drugs based on therapeutic categories (*n* = 759)

Therapeutic Categories of Drugs	n(%)
Antidepressants	35(4.6)
Antidiabetics	58(7.6)
Antihypertensives	103(13.6)
Antimicrobials	153(20.2)
Lipid- lowering Drugs	23(3.0)
Hypnotics and sedatives	18(2.4)
Antipsychotics	15(2.0)
Corticosteroids	16(2.1)
Vitamins and minerals	65(8.5)
Anti-cold and Cough preparations	13(1.7)
Anticoagulants	5(0.7)
Analgesic, Anti-inflammatory Drugs and Antipyretics	55(7.2)
Antiepileptics	12(1.6)
H_1_-antihistamines	32(4.2)
Proton-pump inhibitors	27(3.6)
Antiemetics	9(1.2)
Antispasmodics	15(2.0)
Bronchodilators and Anti-asthma Drugs	14(1.8)
Muscle relaxants	14(1.8)
Antigout Drugs	7(0.9)
Tropical Nasal Decongestants	5(0.7)
Miscellaneous	65(8.6)

Primary labeling of all pharmaceutical products had the name of the drug (brand); however, only over three-quarters of products had stated the quantity of the drug. Similarly, almost all the products declared the category of the drug while only a few products (4.1%) mentioned the sub-category. The system of medicines was stated in around one out of 10 products. Active ingredients and their quantity were mentioned in almost all products. But, 11% products lacked the name of pharmacopoeia belonging to the drug. None of the products mentioned serial number for establishment of pharmaceutical industry. Almost all products stated drug production company, address and country, serial number for production of drug, date of production, storing methods [but keep out of reach of children was not mentioned in 46(6%) products] and information on quantity used. Similarly, all products had the batch number and the date of expiry. However, five out of 100 products did not list their selling price. Few Group “a” and Group “b” products (2.4%) lacked caution labeling (not to be sold without the prescription of a registered medical practitioner). More importantly, approximately seven percentage of products were found to provide misleading information with respect to caution [example: stating ‘not to be sold without the prescription of a registered medical practitioner’ in case of OTC drugs (Group “c”)]. Unfortunately, majority of products (84.4%) did not provide information on directions of use. The appropriate drug classification was found in 89.6% of products (Table 3).

**Table 3:** Information mentioned in primary labeling of medicines (*n* = 759)

Information	Categories	n(%)
Drug name (Brand)	Yes	759(100)
Drug quantity	Yes	583(76.8)
Category of drug	Yes	757(99.7)
Sub-category of drug	Yes	31(4.1)
System of medicine	Yes	75(9.9)
Active ingredient and quantity	Yes	758(99.9)
Pharmacopoeia	Yes	676(89.1)
Drug production company, address and country	Yes	758(99.9)
Serial number for establishment	Yes	0(0)
Serial number for production	Yes	757(99.7)
Batch number	Yes	759(100)
Date of production	Yes	752(99.1)
Date of expiry	Yes	759(100)
Price	Yes	719(94.7)
Storage	Yes	759(99.9)
*Caution in Group “a” and “b” (not to be sold without the prescription of a register medical practitioner)	Yes	588(77.5)
	No	18(2.4)
	Misleading information	52(6.9)
	Not applicable (Category c)	101(13.3)
Quantity to be used	Yes	743(97.9)
Method of use	Yes	116(15.3)
External preparation (only for external use) (*n* = 55)	Yes	54(98.2)
Appropriateness of drug category	Appropriate	680(89.6)
	Inappropriate	77(10.1)
	Unable to assess	2(0.3)

*Percentage do not add up to 100 due to single decimal

Out of 88 OTC products studied, almost all mentioned the drug name and category of drug in Nepali language as provisioned in the DDA’s requirements. As shown in Table 4, some information were not assessed because they were lacking. While a total of 18.2% OTC products stated directions of use, only 14.8% of products mentioned it in Nepali language. Most of the OTC products (84.1%) mentioned only necessary precautions in Nepali language, but majority of them were found without batch number (84.1%), date of production (83%), date of expiry (84.1%) and price (76.1%) in Nepali language (Table 4).

**Table 4:** Assessments of labeling of OTC (DDA listed) medicines where certain information required to write in Nepali language (*n* = 88)

Information	Categories	n(%)
Drug name (brand) in Nepali language	Yes	87 (98.9)
Method of use in Nepali language	Yes	13(14.8)
	No	3(3.4)
	Unable to assess	69(78.4)
Category of drug in Nepali language	Yes	85(96.6)
Precaution taken in Nepali language	Yes	74(84.1)
	No	13(14.8)
	Unable to assess	1(1.1)
*Price Nepali language	Yes	15(17.0)
	No	67(76.1)
	Unable to assess	6(6.8)
Date of production in Nepali language	Yes	14(15.9)
	No	73(83)
	Unable to assess	1(1.1)
Date of expiry in Nepali language	Yes	14(15.9)
Batch number in Nepali language	Yes	14(15.9)

*Percentage do not add up to 100 due to single decimal

Other labeling errors were also encountered during the assessment of primary labeling. This included duplication of same information in two products (0.3%), while sticker with new price by erasing original price, inappropriate dosage information, incorrect method of use, no printed information (except batch number, date of production, date of expiry and price), erasing of drug category with permanent marker, each of which were observed in different single product (0.1%).

## Discussion

The pharmaceutical sector of Nepal continues to grow, with over 15,000 registered products, over 300 registered manufacturing facilities and over 16,000 pharmacy outlets. This growth demands additional human resources for the regulatory authority (DDA) to improve regulation and smooth functioning of the authority body [15]. As a consequence, the gap between regulatory provision and actual practice has been documented. [8–10]. To keep this in check, primary labeling of medicines should be assessed on a regular basis.

Studies have demonstrated that labeling and packaging are causal factors for medication errors [2, 16–18]. Therefore, appropriate labeling of marketed medicines is necessary for fulfilling the regulatory provision and ensuring patient medication safety [3]. As observed in this study, nearly a quarter of products were lacking information on quantity or strength of the drug. This might be due to difficulty in stating strength along with their brand name in those products containing multiple active ingredients. However, it was observed that almost all products had information on active ingredient(s) and respective quantity to communicate the composition of products. Majority of products had not mentioned sub-category of drugs on their labeling but mandatory presence of this information from the perspective of consumers still remains controversial. Similarly, most of the products were found to lack information on system of medicine they belong to. Practice of categorizing drugs as “a”, “b” or “c” was observed. Pharmacopoeial standard of product, which essentially reflects standard of the product, [19] was missing in some of the labels. Perhaps, this issue should be of serious concern for stakeholders because this is considered important for drug safety and is of special concern for consumers. Drug Standard Regulation, 2043 (1986 AD) mentions that products need to have serial number for establishment of pharmaceutical industries [11]. This provision was found ignored on labelling of all medicines of all drug companies that were assessed. This might be because it is considered less relevant to patients and healthcare professionals. In this study, all products were observed to include storage information, except for 6% of them that did not have information regarding “keep out of reach of children”. Study in Nigeria reported 89.6% pharmaceutical products had adequate storage instructions [5]. However, this study also included products other than requiring prescription (OTC, antiseptics, disinfectants, detergents, household items, insecticides and consumables). Similarly, majority of medicines stated caution (not be sold without the prescription of a registered medical practitioner) (77.5%) and quantity to be used (97.9%) in this study. Printed words were not always in red or orange, and some products were found to lack ‘signal words’ (e.g. caution, warning) in this study. Printing of such words in colors other than red or orange have been known to lower the perceived hazard and higher proportion of compliant behavior, with red conveying it the strongest [20]. Similarly, it has been demonstrated that the presence of a ‘signal word’ increases perceived product hazard compared to its absence [21]. During this study, we also observed caution mentioned in group ‘c’ drugs, which might be misleading. In Nigeria, a lower proportion of pharmaceutical products adequately mentioned warning and dosage instruction on the labeling of pharmaceutical products [5] but we argue that in Nepal products such as household items, insecticides, detergents and consumables do not come under Drug Act, which was included by this study. However, this study has performed a detail investigation on warning including exploration of first aid instructions as well as sign and symptoms of product toxicity and their treatment. Most of the products in this study had no directions of use, which is important to avoid medication errors [22], especially for modified-release products. For instance, labeling “swallow tablet as a whole without breaking or cursing” on controlled released tablets and “tablets should be chewed before swallowing” on chewable tablets are needed to ensure that patients use it in the intended manner. Studies in SriLanka [6] and Malaysia [7] have also reported insufficient labeling of dispensed medicines as required by the regulatory authorities. The study done in SriLanka used WHO manual for household survey, and included both Western and traditional medicine but did not evaluate repackaging by family members and their understanding of medication administration instruction [6]. Similarly, study in Malaysia used simulated client method to explore labeling requirement of dispensed medicine using a case of common cold but limits itself to single simulated case, observer bias and being a pilot study [7]. Therefore, improved assessment and monitoring of primary labeling of marketed medicines is a dire need.

Medication labeling is rarely available in languages other than English, placing patients with limited English proficiency at greater risk of misunderstanding medication instructions and warnings [23–26]. To overcome this, DDA has necessitated to write at least name of the drug, method of use, category/caution, price, date of production, batch number and date of expiry of 20 category “c” drugs in Nepali language. However, this study demonstrated that none of the products under study totally adhered to this requirement. Besides this, the print size of label was also very small to read. Studies have shown an association between the print sizes and medication errors [27, 28]. This might contribute to increased medication errors particularly by the elderly people. Therefore, pharmaceutical industries should consider the use of medium and large print to increase readability [28], at least for OTC medicines. Other alternative may be to include comprehensive, clear drug leaflets in individual packs of OTC medicines as they are frequently self-administered without consultation with healthcare professionals. Moreover, confusion between look-alike products can be reduced by using Tall man (capital) letters to write drug names [29]. In this study, labeling errors such as the use of sticker with new price to cover the original price, duplication of same information, and so on were also observed. Different countries have their own regulatory provisions for labeling of pharmaceuticals products, because of which, it was not completely compatible with labeling requirement of other countries [4]. Therefore, foreign manufacturers should also comply with labeling requirements before registration of their product in the country of their interest.

Issues on such labeling errors should be removed through strict assessment of medicine labeling by the DDA and also by stakeholders (Manufacturers, Importers and pharmacy outlets). To overcome this, DDA has implemented Drug administration and management system (DAMS) software and warranted all manufacturers to put detailed information of the products in the software before registration/renewal of their products. Additionally, some sort of hologram, for example, as issued by regulatory authority itself, can also be conceptualized to remove counterfeits related to labels.

This study is the first of its kind in Nepal that assessed the scenario of primary labeling inadequacy and suggests stakeholders to rectify them. This is also useful to identify counterfeit and falsified medicines from true ones, which is an overwhelming problem in Nepal. However, this study has certain limitations that should be taken into account while approaching its findings. Limited number of products from only one system of medicine and from single pharmacy outlet were assessed. Also, this study does not cover factors influencing primary labeling of medicine manufactured by Nepalese pharmaceutical industries.

The results of this study highlight the need for stringency in assessment and evaluation of labels of medicines prior to their approval. Further, the regulatory authority need to closely inspect those approved labels for compliance to the provisions during periodic inspections. The Nepalese pharmaceutical industries should also improve their labeling standards/requirements as a part of GMP compliance. Therefore, all stakeholders should take into consideration that improving labeling requirements will eventually improve patients’ safety, products’ quality and provides thorough information like usage and storage requirements.

## Conclusions

The present study demonstrates a gap between regulatory provisions for primary labeling of Nepalese pharmaceutical products and their actual labeling in medicines. It highlights the need for continued monitoring, reassessment and improvement of labeling of pharmaceutical products manufactured by Nepalese pharmaceutical industries.

## Abbreviations

CMCTH: Chitwan Medical College Teaching Hospital
DAMS: Drug administration and management system
DDA: Department of Drug Administration
OTC: Over-the-counter
SPSS: Statistical Package for social sciences
